# Probing intramolecular vibronic coupling through vibronic-state imaging

**DOI:** 10.1038/s41467-021-21571-z

**Published:** 2021-02-24

**Authors:** Fan-Fang Kong, Xiao-Jun Tian, Yang Zhang, Yun-Jie Yu, Shi-Hao Jing, Yao Zhang, Guang-Jun Tian, Yi Luo, Jin-Long Yang, Zhen-Chao Dong, J. G. Hou

**Affiliations:** 1 Hefei National Laboratory for Physical Sciences at the Microscale and Synergetic Innovation Center of Quantum Information and Quantum Physics, University of Science and Technology of China, Hefei, Anhui China; 2 Department of Chemical Physics and School of Physics, University of Science and Technology of China, Hefei, Anhui China; 3 State Key Laboratory of Metastable Materials Science and Technology & Key Laboratory for Microstructural Material Physics of Hebei Province, School of Science, Yanshan University, Qinhuangdao, China

**Keywords:** Single-molecule fluorescence, Fluorescence spectroscopy, Scanning probe microscopy

## Abstract

Vibronic coupling is a central issue in molecular spectroscopy. Here we investigate vibronic coupling within a single pentacene molecule in real space by imaging the spatial distribution of single-molecule electroluminescence via highly localized excitation of tunneling electrons in a controlled plasmonic junction. The observed two-spot orientation for certain vibronic-state imaging is found to be evidently different from the purely electronic 0–0 transition, rotated by 90°, which reflects the change in the transition dipole orientation from along the molecular short axis to the long axis. Such a change reveals the occurrence of strong vibronic coupling associated with a large Herzberg–Teller contribution, going beyond the conventional Franck–Condon picture. The emergence of large vibration-induced transition charges oscillating along the long axis is found to originate from the strong dynamic perturbation of the anti-symmetric vibration on those carbon atoms with large transition density populations during electronic transitions.

## Abstract (web summary)

Vibronic coupling is a key feature of molecular electronic transitions, but its visualization in real space is an experimental challenge. Here the authors, using scanning tunneling microscopy induced luminescence, resolve the effect of vibronic coupling with different modes on the electron distributions in real space in a single pentacene molecule.

## Introduction

Vibronic coupling in a molecule involves the interaction between electronic and nuclear motions, which is a central issue in molecular electronic transitions and molecular spectroscopy^1–9^. Since low-mass electrons move much faster than the heavy nuclei, the electrons are expected to respond almost instantaneously to the displacement of nuclei, and under Born–Oppenheimer approximation, the motions of electrons and nuclei in a molecule can be treated separately^5^. When a molecule is undergoing an electronic transition, according to the Franck–Condon (FC) principle, the nuclear framework is considered to be stationary and the intensity of resultant vertical vibronic transition is proportional to the square of the overlap integral between the vibrational wavefunctions of the excited and ground states. Nevertheless, in reality, the dynamic motions of nuclei (i.e., molecular vibrations) can modify the electronic wavefunctions of the excited or ground states so strongly as to trigger vibration-induced emission that is usually forbidden based on the FC principle^1,6,7^. Therefore, a comprehensive investigation on the intramolecular vibronic coupling needs to go beyond the traditional FC picture and take the Herzberg–Teller (HT) contribution into account^4,8^. However, due to the diffraction limit in conventional far-field optics, it is highly challenging to visualize the real-space feature of vibronic coupling within a single molecule. The microscopic picture of how molecular vibrations affect electronic transitions remains to be addressed.

Scanning tunneling microscope (STM) induced luminescence (STML) enables spectroscopic imaging with sub-nanometer resolution beyond the diffraction limit thanks to the highly localized nature of tunneling electron excitations and nanocavity plasmon (NCP) enhancement^10–18^. Such a technique has demonstrated its power in exploring vibrationally resolved spectroscopy^10–13,16,18–21^, even vibronic maps for molecular trimers^20^. However, despite these advances, several fundamental questions on the vibronic coupling within a single molecule still remain to be clarified. First, what are the real-space features of vibronic transitions within a single molecule? Second, what kind of molecular vibrations will impose strong perturbation on the electronic transition to generate intense vibration-induced emission? Third, more importantly, how exactly a specific molecular vibration affects the electronic transition in real space?

In this paper, we address all these issues with STML spectroscopic imaging by using an anisotropic linear pentacene molecule as a model system. We reveal distinct real-space features of vibronic coupling within a single molecule through sub-nanometer resolved spectroscopic imaging. By combining with theoretical calculations, we provide a microscopic picture on why the HT-dominated vibronic emission could have a differently oriented transition dipole from that of the purely electronic transition and how a specific vibration affects electron distributions in real space within a single molecule.

## Results

### Vibronically resolved electroluminescence from a single pentacene molecule

**Fig. 1:**
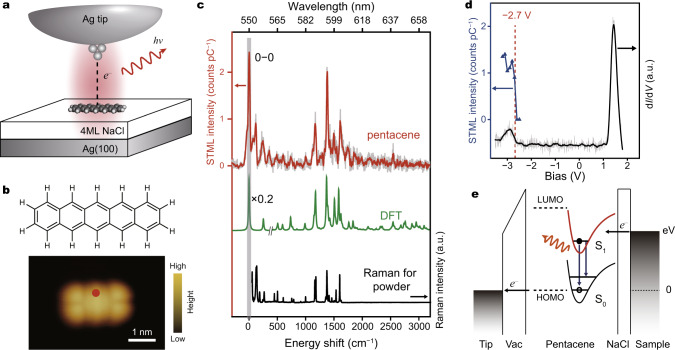
Vibronically resolved electroluminescence from a single pentacene molecule. **a** Schematic of the STML experiment on a single pentacene molecule on NaCl/Ag(100). The red halo refers to the nanocavity plasmon (NCP). Molecular fluorescence is generated by the excitation of highly localized tunneling electrons over a single pentacene molecule that is decoupled by NaCl layers from the Ag(100) substrate. **b** STM image of a single pentacene molecule adsorbed on 4ML-NaCl/Ag(100) (5 × 3.5 nm^2^; −3 V, 2 pA), with the molecular structure shown on the top. The color scale represents the topographic height and applies to all the STM images presented in this work. **c** Typical STML spectrum (−3 V, 2 pA, 60 s) acquired at the red point marked in **b**, showing rich vibronic emission peaks. Also plotted are a DFT simulated fluorescence spectrum from a single pentacene (green curve) and a Raman spectrum acquired from a pentacene powder sample (excitation laser wavelength: 785 nm) (black curve). **d** Typical d*I*/d*V* spectrum acquired at the red point marked in **b**. The setpoint for the d*I*/d*V* measurement was −3.5 V and 5 pA. The integrated molecular emission intensities at different excitation voltages are also shown (blue curve and filled triangles). **e** Schematic of the carrier-injection excitation mechanism for the molecular electroluminescence. A singlet exciton is generated through two sequential carrier injection steps. Then, the excited molecule decays radiatively from the excited state (S_1_) to the ground state (S_0_). The raw data are plotted in gray lines in **c** and **d**.

To probe intramolecular vibronic coupling, one of the desirable approaches is to obtain single-molecule emission spectra as they often contain rich vibronic features^2,10,12,21–24^. However, in spite of several previous reports on the electroluminescence from pentacene nanocrystals^25,26^, the demonstration of single-molecule electroluminescence from an isolated pentacene is not trivial due to the low quantum efficiency (∼0.08)^27^ and poor anchoring stability of the molecule on the dielectric surface. Therefore, in order to realize the electroluminescence from a single pentacene in the STM junction, we adopted a combined strategy of effective electronic decoupling and strong nanocavity plasmon enhancement, as illustrated schematically in Fig. 1a. The former is achieved by using a 4-monolayer (ML) thick NaCl island as a dielectric spacer to separate the pentacene molecule from the underlying Ag(100) substrate so that substrate-induced fluorescence quenching can be effectively suppressed. The latter is realized by fine-tuning the Ag tip status to achieve strong NCP enhancement (detailed in Supplementary Note 1). Such a strong plasmonic enhancement is particularly critical for the present study, because, to prevent the diffusion or damage of pentacene molecules during the STML measurements, very low currents (e.g., down to 2 pA) have to be used due to the very poor conductivity of 4-ML NaCl. If without strong NCP enhancement, the signal-to-noise ratio under such a low excitation power would be too poor to yield meaningful emission spectra, not to mention spectroscopic imaging.

Figure 1b shows the STM image of a single pentacene molecule adsorbed on 4-ML NaCl on Ag(100) acquired at negative bias, which is dominated by the features of the highest occupied molecular orbital (HOMO), though with a slightly poorer resolution compared to that reported previously using a tungsten tip^28^. When the tip is positioned above the upper-middle position of the single pentacene along the short axis direction, as indicated by the red dot in Fig. 1b, we obtain an electroluminescence spectrum with sharp multi-peak features (red curve in Fig. 1c). The peak with the highest emission energy is at ~549.7 nm, which is assigned to the 0–0 emission peak of a single pentacene by referring to the 0–0 peak energy of ~536.9 nm reported in the photoluminescence measurements on pentacene-rare gas complexes^29^, though red-shifted by as large as ∼54 meV because of the relatively stronger interaction of pentacene with the NaCl substrate or plasmonic nanocavity. Such an assignment is also supported by the agreement with the calculated emission spectrum (detailed in Supplementary Note 2), as shown in Fig. 1c. Upon such an assignment of the 0–0 peak, the vibronic peaks (i.e., representing vibronic states) with lower energies in the STML spectra can be expressed as the frequency shifts with respect to the band origin (i.e., the 0–0 peak)^20,21^. Strikingly, the vibronic peaks in the STML spectrum are very similar to those in the Raman spectrum measured on a powder sample (black curve in Fig. 1c). Such an agreement not only gives solid experimental justification about the assignment of the 0–0 peak, but also allows to correlate the vibronic states present in the emission with specific molecular vibrational modes in Raman spectra.

The observation of many vibronic peaks in Fig. 1c indicates that the transitions between the excited state and ground state are not purely electronic transitions, but rather vibronic transitions involving intramolecular interactions between electrons and vibrations. Before we further discuss on the intramolecular vibronic coupling, let us look at the excitation mechanism of the electroluminescence first to see what kind of molecular orbitals or states are involved during the electronic transition process. Figure 1d shows the differential conductance (d*I*/d*V*) data over a single pentacene (black curve). The HOMO state starts to appear at about −2.7 V and the lowest unoccupied molecular orbital (LUMO) state emerges at ~1.2 V, consistent with the previous report^28^. Notably, a sharp rise in the molecule-specific emission intensity (blue curve in Fig. 1d) is also observed at the sample bias voltage of about –2.7 V (detailed in Supplementary Note 3), which coincides with the threshold energy to extract electrons from the HOMO state. Such a coincidence suggests a carrier-injection model as the dominant excitation mechanism, with the inelastic electron scattering mechanism playing a negligible role^14,30^. Thus, the picture for the excitation of the single-molecule electroluminescence here can be understood as follows through two sequential carrier-injection steps (Fig. 1e): first, when the HOMO state of the pentacene molecule is raised above the Fermi level of the tip by a negative external voltage, an electron in the HOMO tunnels to the tip, leaving a hole behind in the molecule (in other words, a hole is injected into the HOMO); second, another electron in the substrate can be injected into the empty state of the transient pentacene cation whose energy is significantly lowered due to attractive Coulomb interactions, thus bringing the molecule back to the excited neutral state S_1_ (namely, forming a singlet exciton). Finally, accompanying the electronic transition from the LUMO to HOMO state, the excited molecule decays radiatively from the excited state (S_1_) to the ground state (S_0_), with the de-excitation rate greatly enhanced by the nanocavity plasmon.

### Spatially and spectrally resolved vibronic-state imaging

**Fig. 2:**
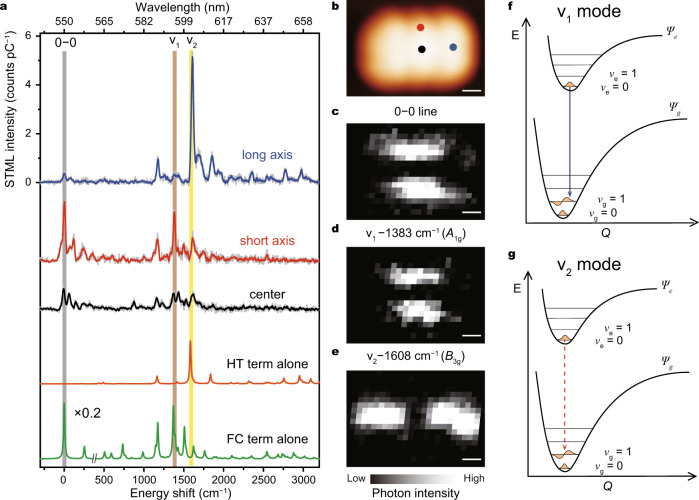
Position-dependent STML spectra and spectroscopic imaging for selected vibronic peaks. **a** Three typical STML spectra (−3 V, 2 pA, 60 s) acquired at the positions marked in **b**. The raw data are plotted in gray lines. The calculated spectra for the contributions from either Franck–Condon term (green curve) or Herzberg–Teller term (orange curve) alone are also plotted. The shaded bands labeled as 0–0, v_1_ and v_2_ highlight the corresponding peaks. **b** STM image of a single pentacene molecule on 4ML-NaCl/Ag(100) (3.7 × 2.6 nm^2^; −3 V, 2 pA). **c**–**e** Energy-resolved spectroscopic images for the 0–0 peak (0 ± 7 cm^−1^) (**c**), the v_1_ mode (1383 ± 6 cm^−1^) (**d**), and the v_2_ mode (1608 ± 6 cm^−1^) (**e**). Imaging condition: 3.7 × 2.6 nm^2^; −3 V, 2 pA; 10 s per pixel. **f**, **g** Schematics of the potential energy curves for the v_1_ mode (**f**) and the v_2_ mode (**g**) based on the DFT calculations. Scale bars, 0.5 nm.

The NCP is crucial not only for amplifying the radiative decay rate, but also for coupling the different molecular emissions in the junction to the far field as detectable photons. By investigating the dependence of STML spectra on different excitation positions over a single molecule, we can explore the angular dependency of plasmon−exciton coupling^19,21,31^, which can be used to reveal the orientation of transition dipole for each vibronic state and thus gain insights into how molecular vibrations are coupled with electronic transitions. As shown in Fig. 2a for three representative STML spectra, the energies of different vibronic states remain almost the same for different excitation positions marked in Fig. 2b, but their relative emission intensities vary a lot, as exemplified by the 0–0 peak and other two strong vibronic peaks labeled as v_1_ and v_2_. To be specific, the v_1_ peak at ~594.7 nm (associated with the vibrational mode at 1383 cm^–1^) is found to be very strong when excited at the short axis (e.g., the red point in Fig. 2b) and becomes much weaker when excited at the long axis (e.g., the blue point in Fig. 2b), in parallel with the intensity evolution of the purely electronic 0–0 peak at ~549.7 nm. By contrast, an opposite position-dependent behavior is observed for the v_2_ peak at ~602.9 nm (associated with the vibrational mode at 1608 cm^–1^), where the emission is found to be very strong when excited at the long axis but becomes weak at the short axis. Such a distinctly different behavior of the v_2_ emission from the purely electronic 0–0 peak implies a strong perturbation of the v_2_ vibrational mode on the electronic transition. Note that all these emission peaks become weak when the tip is positioned above the pentacene center owing to the dipole symmetry of the whole system during STML measurements^14,19^.

In order to get a panoramic view on the spatial distributions of different vibronic states so that the influence of molecular vibrations on the transition dipole orientations can be directly visualized, we carried out spatially resolved spectroscopic imaging over a single molecule^14,20^, which is a highly challenging experiment for the pentacene molecule due to its poor quantum efficiency and low anchoring stability on the surface. The pentacene molecules adsorbed on 4ML-NaCl are found to be optimal and feasible for carrying out such spectroscopic imaging experiments. As shown in Fig. 2c−e, the imaging patterns for the 0–0, v_1_, and v_2_ emissions all exhibit a two-spot pattern but with different orientations. The two-spot patterns for the 0–0 and v_1_ peaks are both along the short axis, indicating their stronger emission behavior when excited along the short axis. However, for the v_2_ peak, the two-spot pattern rotates 90° with respect to that for the purely electronic 0–0 emission and becomes oriented along the molecular long axis, which is quite remarkable and suggests a strong vibronic coupling for the v_2_ vibrational mode. According to previous studies^14,19^, the two-spot pattern directly reflects the orientation of molecular transition dipole moment. Therefore, based on the observed patterns of spectroscopic images presented in Fig. 2c−e, we can know that the transition dipole is oriented along the molecular short axis for the purely electronic 0–0 and vibronic v_1_ transitions, but strikingly becomes oriented along the molecular long axis for the v_2_ transition. In other words, the v_2_ vibration strongly perturbs the electronic transition, indicating the occurrence of strong vibronic coupling. It should be noted that only those transitions with transition-dipole orientations aligned with the radially polarized electric field of NCP can be efficiently enhanced^19,21,31^. Thus, the v_1_ (v_2_) mode with its transition dipole along the short (long) axis can be efficiently enhanced when exciting at the short (long) axis. Such a selective NCP enhancement is probably critical for the observation of a clear v_2_ peak in our experiment, since such a vibronic peak involving HT contributions (detailed below) is usually difficult to measure in conventional far-field optical spectroscopy^22,32^.

In order to further understand the origin of the vibronic peaks and associated imaging patterns, let us look at some theoretical backgrounds about electronic transitions. Vibronic coupling is intrinsically a quantum process where vibronic and electronic degrees of freedom are intertwined. But in the practical calculation of molecular spectra, semi-classic approaches are often adopted to treat vibronic coupling under the Born–Oppenheimer approximation to allow the separation of electronic and nuclear coordinates. In this case, as detailed in Supplementary Note 4, section 4.1, the electronic transition dipole for different vibronic peaks can be calculated by treating the vibration as a perturbation^2,20,33,34^ and expressed as:

1 $${\mathbf{\upmu }}_{{\mathrm{eg}}} = {\mathbf{\upmu }}_{{\mathrm{eg}}}(Q_0)\left\langle {v_{\mathrm{g}}|v_{\mathrm{e}}} \right\rangle + \mathop {\sum}\limits_k {\left( {\frac{{\partial {\mathbf{\upmu }}_{{\mathrm{eg}}}}}{{\partial Q_k}}} \right)_{\mathrm{0}}\left\langle {v_{\mathrm{g}}|Q_k|v_{\mathrm{e}}} \right\rangle } ,$$

where *v*_e_ (*v*_g_) represents the nuclear vibrational wavefunction of excited state (ground state), **μ**_eg_(*Q*_0_) represents the static transition dipole of the molecule at the equilibrium geometry *Q*_0_, *Q*_*k*_ represents the normal coordinate for the *k*-th vibration of the molecule.

The first term in Eq. (1) refers to the FC term, which describes the vertical electronic transition within a stationary nuclear framework and usually makes dominant contributions to dipole-allowed vibronic transitions involving total symmetric vibrations. The FC term stems from the same electronic states of the 0–0 purely electronic transition (**μ**_*eg*_(*Q*_0_)) and is proportional to the overlap integral of the vibrational wavefunctions (i.e., the FC factor). According to the selection rule^2,18^, an electronic transition is dipole-allowed only when the Kronecker product of the transition, Γ_e_⊗Γ_d_⊗Γ_g_ contains the totally symmetric representation, in which Γ_g_, Γ_e_, and Γ_d_, represent the irreducible representation of the electronic ground state, excited state, and the dipole operator. The second term in Eq. (1) refers to the HT coupling term, which can come into play only when the Kronecker product, Γ_e_⊗Γ_d_⊗Γ_*g*_⊗Γ_v_ contains the totally symmetric representation after considering the additional symmetry of the vibration Γ_v_. Such a HT term describes the dynamic influence of molecular vibrations on electronic transitions and offers the understandings on vibronic coupling beyond the commonly used FC picture. Based on the theoretical framework above, the identical transition-dipole orientation of the v_1_ mode to the 0–0 peak observed experimentally, as illustrated in Fig. 2c, d, suggests the dominant contribution of the FC term to the v_1_ peak. More importantly, the change of transition-dipole orientation for the v_2_ mode with respect to 0–0 peak indicates a dominant contribution of the HT term to the v_2_ peak and thus reveals clearly the dynamic spatial influence of the v_2_ vibrational mode on the electronic transition through a strong vibronic coupling. It is worth noting that such an understanding on the spatial dependency of vibronic peaks is similar to that proposed in a previous study for the phthalocyanine molecular trimer^20^.

These assignments for the dominant contributions to the vibronic transitions are also substantiated by the DFT calculations on the emission spectra that show either the FC or HT term alone (see more details in Supplementary Note 4, section 4.2). As illustrated in Fig. 2a by the green and orange curves, the v_1_ peak is indeed dominated by the FC term while the v_2_ peak is dominated by the HT term. These calculations also provide important information on the change of the equilibrium nuclear configurations during vibronic transitions, as shown in Fig. 2f, g. Specifically, the v_1_ vibronic transition undergoes evident displacement between the minima of the potential energy curves for the excited and ground states, leading to a large FC contribution based on the Franck–Condon principle. However, surprisingly, the v_2_ vibronic transition experiences almost no displacement for the potential energy curves, suggesting very small FC factors and negligible FC contributions, thus making the v_2_ peak a strong vibration-induced emission. The observation of such an emission is indicative of a strong dynamic perturbation of the v_2_ vibration on the electronic transition.

### Theoretical analysis on vibronic coupling and vibration-induced emission

As revealed in Fig. 1e for the carrier-injection excitation mechanism, the electroluminescence is associated with the LUMO–HOMO transition. In order to gain more insights into the coupling between the electronic transition and molecular vibration in real space, we also performed DFT calculations on the spatial distributions of pentacene HOMO and LUMO states accompanying the transition as well as vibration-induced transition charges (detailed in Supplementary Note 4, sections 4.3 and 4.4). During an electronic transition from LUMO to HOMO, the bonding and anti-bonding characters between atoms will vary, and as a result, the nuclei are subjected to a change in Coulombic forces due to the redistribution of electronic charge (detailed in Supplementary Note 4, section 4.3). Thus, the nuclei will respond through vibrations, leading to the appearance of vibronic peaks in emission spectra^1^. The question is what kind of molecular vibrations will impose strong perturbations on the electronic transition to generate intense vibration-induced emission? And how do they affect electronic transitions?

**Fig. 3:**
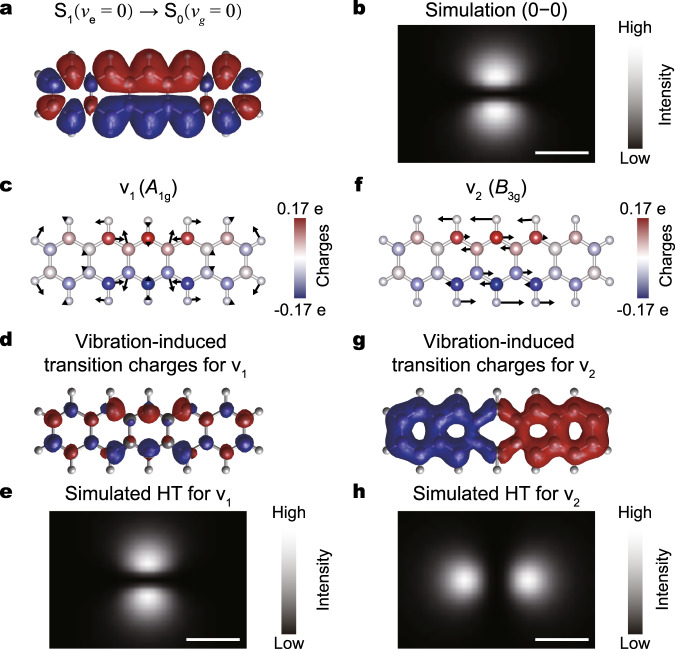
Simulations on transition densities and vibration-induced transition charges. **a** Simulated transition densities for the S_1_ → S_0_ transition. The blue and red colors indicate the spatial distributions of the positive and negative charges. **b** Simulated photon image for the 0–0 transition. **c**, **f** Schematics of the vibrations for the v_1_ and v_2_ modes, superimposed with the S_1_ → S_0_ transition densities distributed over each atom by Hirschfield population analysis. **d**, **g** Calculated vibration-induced transition charges for the v_1_ and v_2_ modes. **e**, **h** Simulated photon images of the HT-term contributions for the v_1_ and v_2_ modes. Scale bars, 1 nm.

In order to address these issues, we calculate the spatial distribution of the purely electronic transition dipole (S_1_ → S_0_), which is approximated by the transition densities through the convolution of LUMO and HOMO, as shown in Fig. 3a. There are two primary features. The first one is that the simulated transition charge oscillation is oriented along the short axis, which leads to a simulated two-spot pattern for photon imaging also along the same direction^35^ (Fig. 3b). The second feature is that the transition density are dominantly populated over the six central carbon atoms on the two sides (Fig. 3a, c, d), which reflects the spatial distribution of the electronic transition probabilities. Such a distribution may explain why the v_1_ and v_2_ peaks are relatively strong among all the vibronic peaks since these two vibrations perturb strongly to these six carbon atoms (see Supplementary Movie 1 for more details).

As illustrated in Fig. 3c, for the v_1_ mode with *A*_g_ symmetry, although all the atoms are moving more or less, the six central carbon atoms and part of their nearest-neighbor atoms vibrate even harder, expanding and shrinking in a total symmetric way (Supplementary Movie 1). Such a symmetric motion is found to generate a strong FC term that has the same symmetry as the 0–0 transition. Nevertheless, the HT term caused by the v_1_ vibration is found to be negligible, as illustrated in Fig. 3d through the very small amount of vibration-induced transition charges (detailed in Supplementary Note 4, sections 4.5 and 4.6). Because of the *A*_g_ symmetry of the v_1_ vibrational mode, the induced transition charges oscillate along the short axis, leading to a two-spot pattern along the short axis (Fig. 3e). But it should be borne in mind that this HT term contributes little to the photon image of the v_1_ mode experimentally observed, which is dominated by the FC term.

However, as illustrated in Fig. 3f, for the anti-symmetric v_2_ mode with *B*_3g_ symmetry, the top and bottom six carbon atoms oscillate in an opposite manner along the long axis and with evidently large amplitudes relative to other atoms (see Supplementary Movie 1 for details). Such a vibration breaks the molecular symmetry and can induce a strong vibronic coupling. For the v_2_ vibration, the dipole-allowed FC component is still along the short axis, but its contribution is negligible due to the almost unchanged equilibrium nuclear configurations (Fig. 2g). However, due to the HT coupling or contribution, the original dipole-forbidden transition along the long axis becomes allowed, because the corresponding Kronecker product, Γ_e_⊗Γ_d_⊗Γ_g_⊗Γ_v_, now contains the totally symmetric representation due to the coupling with the v_2_ vibration of *B*_3g_ symmetry (see Supplementary Note 4, section 4.6 for detailed symmetry analysis). As illustrated in Fig. 3g, the perturbation of the v_2_ vibration on the electronic transition is so strong that it creates large transition charges that are oscillating along the long axis, which can be efficiently enhanced by the NCP when the tip is positioned at the long axis. Naturally, the photon imaging pattern simulated based on such transition charge oscillation is along the long axis (Fig. 3h) (detailed in Supplementary Note 4, section 4.5). This is the primary reason why the observed two-spot pattern for the v_2_ mode (Fig. 2e) is oriented along the long axis. The distinctly different pattern of the v_2_ peak from the purely electronic 0–0 transition indicates directly its vibration-induced emission nature. It should be noted that such vibration-induced emission is often discussed in the literature using an intensity borrowing mechanism via the state mixing with other high-lying eigenstates^18,20,36^, a quantum and more rigorous formulation. In the present situation, the v_2_-vibration induced emission most probably borrows the contribution from the S_1_ → S_2_ transition in terms of the analysis on the dipole orientations and energy differences (detailed in Supplementary Note 4, section 4.7). In other words, the v_2_ vibration with *B*_3g_ symmetry is likely to modulate the zero-order electronic wavefunction of the S_0_ state in a way to best resemble that of the S_2_ state (i.e., induce efficient mixing of the electronic ground state S_0_ with the electronic excited state S_2_), so that the v_2_-vibration induced emission seems to borrow intensities from neighboring electronic transitions.

### Vibronic emission and imaging patterns of perdeuterated pentacene

**Fig. 4:**
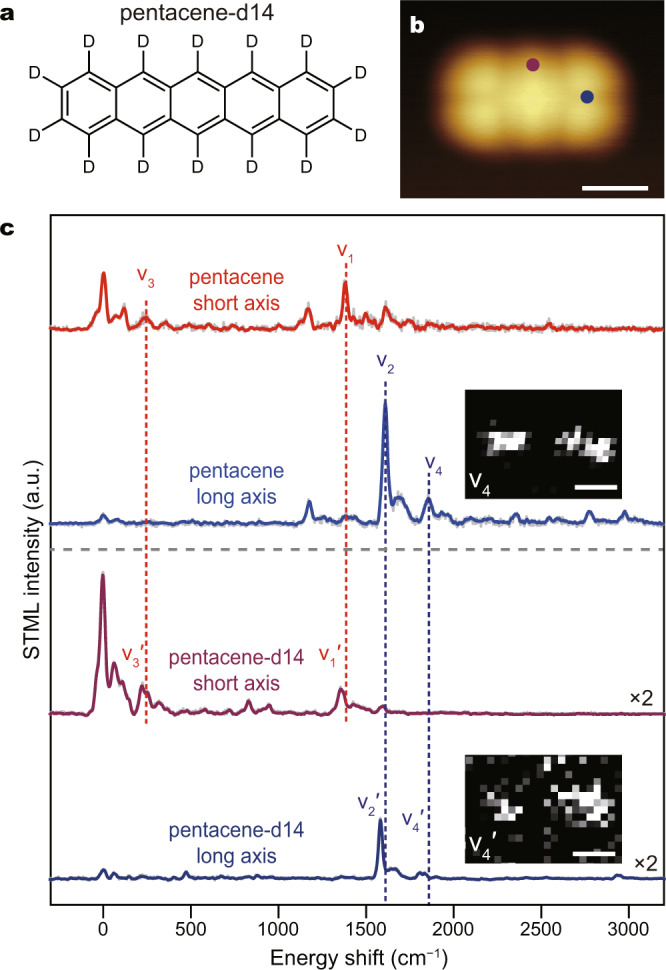
STML measurements on a single perdeuterated pentacene molecule. **a** Molecular structure of perdeuterated pentacene (noted as pentacene-d14). **b** STM image of a single pentacene-d14 molecule adsorbed on 4ML-NaCl/Ag(100) (4 × 3.5 nm^2^; −3 V, 2 pA). **c** Typical STML spectra from a perdeuterated pentacene molecule acquired at the positions marked in b (−3 V, 2 pA, 60 s), plotted as energy shifts with respect to the 0–0 electronic origin. The raw data are plotted in gray lines. The corresponding STML spectra from a pentacene molecule are also plotted for comparison. The insets show the energy-resolved spectroscopic images (3.7 × 2.6 nm^2^; −3 V, 2 pA) for the v_4_ mode (1855 ± 5 cm^−1^) of a single pentacene molecule (10 s per pixel) and the v_4_′ mode (1806 ± 5 cm^−1^) of a perdeuterated pentacene molecule (5 s per pixel), respectively. Scale bars, 1 nm.

In order to further illustrate the influence of molecular vibrations on electronic transitions, we also carry out STML experiments on isotope substituted pentacene molecules (i.e., perdeuterated pentacene) (Fig. 4a−c) because both types of pentacenes have the same electronic configurations but different vibrational frequencies, with the latter quantities being known to be inversely proportional to the square root of reduced mass. The perdeuteration of pentacene is found to slightly blue-shift the 0–0 electronic origin by ∼33 cm^−1^ (from 549.7 to 548.7 nm) due to the heavy-atom substitution effect^22^ (detailed in Supplementary Note 5). As expected, the respective vibronic emission peaks are blue-shifted slightly stronger upon such replacement, which makes the vibrational frequencies for pentacene-d14 all become smaller compared with their counterpart modes for pentacene, as exemplified for v_1_′ in Fig. 4c, though with different amplitudes (e.g., red-shifted by ∼48 cm^−1^ for v_1_ and by ∼26 cm^−1^ for v_2_). Since the isotope substitution does not change the spatial distributions of either the electronic transition densities or the molecular vibrations, the transition dipoles and the associated imaging patterns of the vibronic modes for pentacene-d14 are identical to those of their counterpart modes for pentacene, as exemplified by the 0–0, v_1_′, and v_2_′ modes in Supplementary Note 5.

More importantly, the isotopic effect can be used to conclusively assign the vibrational modes for pentacene, especially for the overtone vibrations (>1700 cm^–1^) that are not yet addressed in Fig. 2a, thus enabling a clear investigation about the combined influence of two simultaneously excited fundamental vibrations on the electronic transition. As an example, the v_4_ mode (~1855 cm^–1^) in Fig. 4c for pentacene can be unambiguously assigned to a combination tone of the HT-dominated v_2_ mode (*B*_3g_, 1608 cm^–1^) and the FC-dominated v_3_ mode (*A*_g_, 245 cm^–1^), based on the consideration of sum energy and the consistent shift for its isotope counterpart modes in pentacene-d14, that is, the energy of the v_4_′ mode (1805 cm^–1^) equals to the sum of the vibration energies of the v_2_′ (1582 cm^–1^) and v_3_′ (221 cm^–1^) modes. Notably, the vibronic-state imaging for the v_4_ (v_4_′) mode exhibits a two-spot pattern that is oriented along the long axis, as shown in the inset of Fig. 4c. Such a pattern is very similar to that of the v_2_ peak, which indicates that the combined influence of the simultaneously excited v_2_ and v_3_ modes on the electronic transition is mainly determined by the HT-dominated anti-symmetric v_2_ mode (*B*_3g_), rather than the FC-dominated symmetric v_3_ mode (*A*_g_). Such an understanding, derived directly from imaging patterns, is also supported by the theoretical analysis (detailed in Supplementary Note 4, section 4.8). Consequently, the anisotropic patterns in spectroscopic images offer a straightforward understanding on the microscopic picture of vibronic coupling in real space.

In summary, we have investigated in real space the intramolecular vibronic coupling of a single pentacene molecule through sub-nanometer revolved spectroscopic imaging, by exploiting the localized NCP enhancement on a well-decoupled emitter. The imaging patterns of FC term-dominated vibronic states are found to have the same orientation as that of the 0–0 peak, all along the short axis. However, the patterns of HT term-dominated vibronic states are found to be evidently different from that of the 0–0 peak, becoming rotated 90° along the long axis. Such a difference directly reflects a change in the transition dipole orientation, suggesting the occurrence of strong vibronic coupling associated with a large Herzberg–Teller contribution and going beyond the conventional Franck–Condon picture. By combining with theoretical calculations, the vibration-induced emission is found to occur on those non-total-symmetric molecular vibrations that can strongly perturb the electronic transition, especially through those atoms with large transition density populations. The strong and dynamic vibrational perturbation to these atoms leads to a large vibration-induced transition charges oscillating in a different direction from the purely electronic transition. In this way, anisotropic vibronic-state imaging patterns offer a straightforward understanding on how molecular vibrations affect electronic transitions and related energy redistributions in real space. In addition, we have also investigated the effect of isotope substitution on molecular vibrations. The perdeuteration of pentacene is found to blue-shift both the electronic origin and vibronic emission due to the heavy-atom substitution effect, but vibronic peaks are shifted slightly stronger, yielding the expected frequency red-shifts in respective vibrational modes. Isotope substitution helps to make unambiguous assignments for vibrational modes, particularly for overtone vibrations. Anisotropic vibronic-state imaging for a combination mode composed of one FC-dominated and one HT-dominated fundamental mode indicates that the combined influence of these two simultaneously excited modes on the electronic transition is mainly determined by the HT-dominated mode. Our results provide a profound understanding on the microscopic picture of molecular spectroscopy, particularly on vibronic coupling, and open up new opportunities for real-space studies on the role of electron–vibration coupling in energy transfer processes at the individual molecular level.

## Methods

All STM imaging and STML measurements were performed with a custom low-temperature ultrahigh-vacuum STM (Unisoku) combined with optical detection systems at about 7 K under a base pressure of about 1 × 10^–10^ Torr. The Ag(100) substrate was cleaned by cycles of argon ion sputtering and annealing. Electrochemically etched sliver (Ag) tips were used, which were also cleaned by electron-bombardment and argon-ion sputtering, followed by tip indentations to achieve desired NCP emission modes with strong intensities (tip requirements detailed in Supplementary Note 1). A linear pentacene molecule was used as a model system because of its simple anisotropic structural symmetry (*D*_2h_) and rich optoelectronic properties^37–40^. Pentacene molecules were thermally evaporated onto the Ag(100) substrate partially covered by NaCl islands at ~7 K. STM imaging and optical spectral measurements were performed in a constant-current mode with the sample biased, with the optical setup detailed in our previous reports^14,41^. In order to highlight the vibronic features, the NCP spectral backgrounds in STML spectra and spectroscopic images are subtracted^20^. Differential conductance (d*I*/d*V*) spectra were measured using a lock-in technique with the bias modulation of 10 mV (r.m.s.) at 329 Hz.

## Supplementary information

Supplementary Information

Supplementary Movie 1

Description of Additional Supplementary Files

## Data Availability

The data that support the findings of this study are available from the corresponding authors upon reasonable request.

## Code availability

The codes that support this study are available from the corresponding authors upon reasonable request.
