# Supplementary material for: Probing intramolecular vibronic coupling through vibronic-state imaging
Source: Nat Commun. 2021 Feb 24;12:1280. doi: 10.1038/s41467-021-21571-z (PMC7904785; doi:10.1038/s41467-021-21571-z)
Supplement: Supplementary file 1 — Supplementary Information [file 41467_2021_21571_MOESM1_ESM.pdf]

Supplementary Information

Probing intramolecular vibronic coupling through vibronic-state imaging

Fan-Fang Kong,[1#] Xiao-Jun Tian,[1#] Yang Zhang,[1, 2*] Yun-Jie Yu,[1] Shi-Hao Jing,[1] Yao Zhang,[1,2] Guang-Jun Tian,[3*] Yi Luo,[1, 2] Jin-Long Yang,[1, 2] Zhen-Chao Dong,[1, 2*] J. G. Hou[1]

[1]*Hefei National Laboratory for Physical Sciences at the Microscale and Synergetic Innovation Center of Quantum Information and Quantum Physics, University of Science and Technology of China, Hefei, Anhui 230026, China*

[2]*Department of Chemical Physics and School of Physics, University of Science and Technology of China, Hefei, Anhui 230026, China*

[3]*State Key Laboratory of Metastable Materials Science and Technology & Key Laboratory for Microstructural Material Physics of Hebei Province, School of Science, Yanshan University, Qinhuangdao 066004, China*

Supplementary Note 1. Shaping molecular electroluminescence through tuning the NCP modes

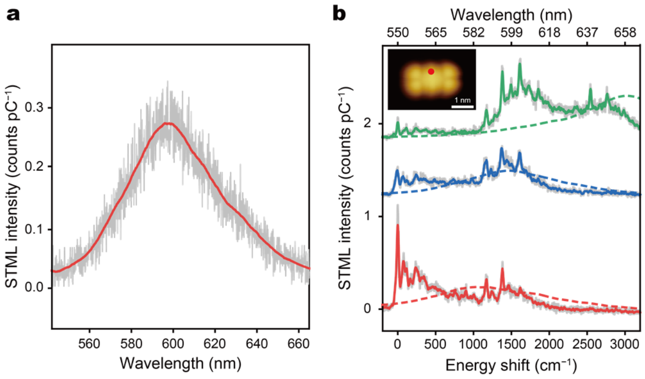

Supplementary Figure 1 | Plasmonic enhancement on the electroluminescence from a single pentacene. a A typical NCP spectrum acquired above 4ML-NaCl/Ag(100) (–3 V, 5 pA, 60 s), with a peak at ~600 nm and a peak intensity of ~0.27 counts pC^{-1}. In order to achieve strong plasmonic

enhancement on the electroluminescence from a single pentacene, Ag tips were used only when the corresponding NCP emission intensity reaches such a typical value or better. **b** The STML spectra over a single pentacene molecule on 4ML-NaCl/Ag(100) acquired with different NCP resonance modes (–3 V, 5 pA, 60 s). The tip position is indicated in the inset STM image marked with a red dot (–3 V, 2 pA). The corresponding NCP emission spectra acquired above 4ML-NaCl/Ag(100) were plotted in dash lines. As illustrated in the figure, the vibronic peaks of a single pentacene can be selectively enhanced by tuning the NCP resonance modes. In the main text, in order to achieve sufficiently strong plasmonic enhancement for spectroscopic imaging experiments and to resonate with various vibronic peaks (including the overtone modes), strongly emitting Ag tips with a peak intensity of ~1.57 counts pC^{-1} and a broad NCP resonance at ~640 nm were adopted for STML measurements on single pentacene molecules on 4ML-NaCl/Ag(100). The raw data are plotted in grey lines in **a** and **b**.

Supplementary Note 2. Comparison of the vibronic peaks obtained from different methods

STML (cm^{-1})	Raman (cm^{-1})	DFT (cm^{-1})
245	241	253
490	500	507
1000	996	987
1170	1177	1174
1383	1372	1369
1496	1501	1508
1608	1598	1583
1855	--	1836
2775	--	2758
2974	--	2952

Supplementary Table 1 | The comparison of the energies for the vibronic peaks obtained from the STML spectrum from a single pentacene, Raman spectrum from a pentancen powder and a DFT calculated emission spectrum, which were displayed in main text Fig. 1c. The vibronic peaks in the STML spectrum were expressed as the frequency shifts (in cm^{-1}) with respect to the band origin (i.e., the 0–0 peak). The Raman spectrum was collected from a pentacene powder sample at room temperature with the excitation laser of 785 nm. The DFT calculation method will be detailed in the subsequent section. Nice agreement could be found among the energies of the vibronic peaks obtained from different methods.

Supplementary Note 3. Voltage-dependent STML spectra from a single pentacene

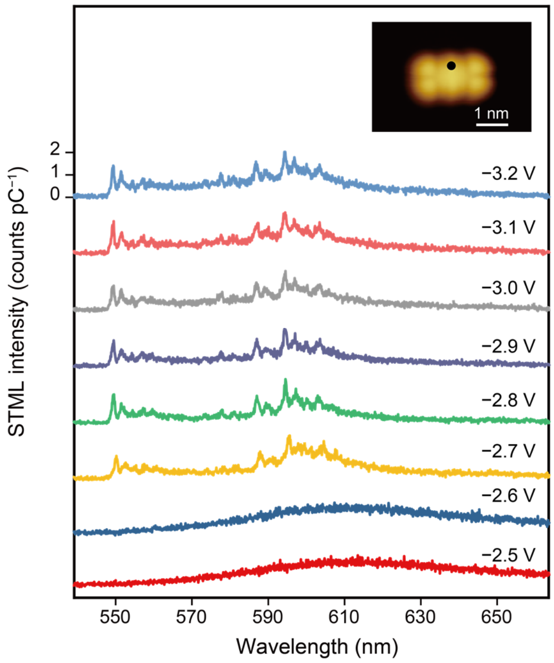

Supplementary Figure 2. | Voltage-dependent emission feature of a single pentacene. STML spectra acquired at different excitation voltages (2 pA, 60 s) over at the position marked with black dot in the inset. The inset of the STM image was acquired at the scanning condition of –3 V and 2 pA. The spectra are vertically shifted for clarity. A sharp rise in molecule-specific emission was observed when the voltage decreases from –2.6 V to –2.7 V, which suggests a threshold voltage of the molecular electroluminescence around –2.7 V. According to the Fig. 1d, the HOMO state starts to appear at about –2.7 V, suggesting the onset energy to extract electrons from the HOMO state. Such a coincidence between the threshold voltage of molecular electroluminescence and the onset of HOMO state implies a carrier-injection model as the dominant excitation mechanism for the STML here, with the inelastic electron scattering mechanism playing a negligible role. We would like to note that the formation of the much lower-energy triplet exciton is also possible through excitations via either inelastic electron scattering[1] or carrier injection[2]. However, since this triplet exciton has a very long lifetime, its quantum efficiency of phosphorescence is believed to be extremely low so that no phosphorescence was observed.

Supplementary Note 4. Descriptions on the theoretical calculations

S4.1 Theoretical background for the vibronic transition

In the framework of the Born-Oppenheimer approximation, the electrons are expected to respond almost instantaneously to the displacement of nuclei and the motions of electrons and atomic nuclei in a molecule can be treated separately[3]. Thus, the wavefunction of a molecular system Ψ can be expressed by the product of the electronic wavefunction ψ and the nuclei wavefunction v. The wavefunction of the ground state (labeled as g) and the excited state (labeled as e) can be expressed as

$$\left|\Psi_g\right\rangle = \left|\psi_g\right\rangle\left|v_g\right\rangle \tag{1a}$$

$$\left|\Psi_e\right\rangle = \left|\psi_e\right\rangle\left|v_e\right\rangle \tag{1b}$$

In order to analyze the vibronic features in electronic transition spectra, people usually adopt the Franck–Condon (FC) principle[4,5], which deals with the electronic transition occurring within a stationary nuclear framework[6]. Thus, based on the FC principle, the transition dipole $\boldsymbol{\mu}_{eg}$ for different vibronic transitions from the excited state (labeled as e) to a ground state can be expressed as:

$$\begin{aligned}\boldsymbol{\mu}_{eg} &= \left\langle\Psi_g\middle|\hat{\mu}\middle|\Psi_e\right\rangle \\ &= \left\langle v_g\middle|\left\langle\psi_g\middle|\hat{\mu}\middle|\psi_e\right\rangle\middle|v_e\right\rangle \\ &\approx \left(\left\langle\psi_g\middle|\hat{\mu}\middle|\psi_e\right\rangle\right)_0\left\langle v_g\middle|v_e\right\rangle \\ &= \boldsymbol{\mu}_{eg}(Q_0)\left\langle v_g\middle|v_e\right\rangle\end{aligned} \tag{2}$$

Where $\boldsymbol{\mu}_{eg}(Q_0)$ represents the electronic transition dipole of the molecule at the equilibrium geometry (Q_0). Under the FC picture, as shown in Eq. (2), the intensity for a vibronic peak is proportional to the vibrational wave function overlap integral. Furthermore, the spatial distributions of the transition dipoles for a vibronic peak should be the same as that for the 0–0 peak, since they all stem from the same electronic states.

The FC principle can well describe the fully-allowed transitions, particularly for those vibronic transitions associated with a molecular vibration of total symmetry[7]. Nevertheless, the dynamic motions of nuclei (i.e., molecular vibrations) can modify the wavefunctions of excited and ground states and mix them with those of other different eigenstates, so that vibronically allowed transitions can be induced that are originally dipole-forbidden based on the FC picture. Therefore, the investigation on vibronic

coupling needs to go beyond the traditional FC picture and take the Herzberg-Teller (HT) contribution into account[8].

In order to include the HT coupling, we need to treat the vibration as a perturbation and consider the first order of Taylor expansion of the transition dipole $\boldsymbol{\mu}_{eg}$ with respect to Q_k (the normal coordinates for the k-th vibration of the molecule)[9]. The transition dipole can thus be expressed as

$$\begin{aligned}\boldsymbol{\mu}_{\mathrm{eg}} &= \langle \Psi_g | \hat{\mu} | \Psi_e \rangle \\ &= \langle v_g | \langle \psi_g | \hat{\mu} | \psi_e \rangle | v_e \rangle \\ &= \left\langle v_g \left| \left(\left(\langle \psi_g | \hat{\mu} | \psi_e \rangle \right)_0 + \sum_k \left(\frac{\partial \langle \psi_g | \hat{\mu} | \psi_e \rangle}{\partial Q_k} \right)_0 Q_k \right) \right| v_e \right\rangle \\ &= \left(\langle \psi_g | \hat{\mu} | \psi_e \rangle \right)_0 \langle v_g | v_e \rangle + \sum_k \left(\frac{\partial \langle \psi_g | \hat{\mu} | \psi_e \rangle}{\partial Q_k} \right)_0 \langle v_g | Q_k | v_e \rangle \\ &= \boldsymbol{\mu}_{eg}(Q_0) \langle v_g | v_e \rangle + \sum_k \left(\frac{\partial \boldsymbol{\mu}_{eg}}{\partial Q_k} \right)_0 \langle v_g | Q_k | v_e \rangle \end{aligned} \tag{3}$$

The first term in in Eq. (3) refers to the FC term, which describes the vertical electronic transition within a stationary nuclear framework and usually produces the dominant contribution for dipole-allowed vibronic transitions involving total symmetric vibrations. The FC term stems from the same electronic states of the 0–0 transition ($\boldsymbol{\mu}_{eg}(Q_0)$) and is proportional to the overlap integral of the vibrational wavefunctions (i.e., the FC factor). The second term in Eq. (3) refers to the HT coupling term, which describes the dynamic influence of molecular vibrations on electronic transitions and offers the understandings on vibronic coupling beyond the commonly-used FC picture.

S4.2 DFT calculation on the spectrum of a single pentacene

Density functional theory (DFT) and time-dependent DFT (TDDFT) calculations were performed to obtain the equilibrium geometry and vibrational frequencies of the pentacene molecule in the ground (S_0) and first excited state (S_1). The B3LYP[10-12] hybrid exchange-correlation function in combination with the 6-31G(d) basis set was applied in all of the DFT and TDDFT calculations. No imaginary frequency was found for either states, which confirms that the optimized geometries are located at the

respective energy minima. The DFT and TDDFT calculations were performed with the Gaussian 16 software[13]. The transition dipole moment between excited states were computed with the quadratic response calculation of the Dalton software[14,15] at the optimized geometry of S_1.

The vibrationally-resolved fluorescence spectra of the pentacene molecule in main text Fig. 1 and Fig. 2 were calculated by computing the FC and HT contributions with a time-independent recursion relationship based on a harmonic approximation as implemented in the DynaVib software[16]. The Duschinsky mode mixing effect was included in the calculations. The derivatives of the transition dipole moment with respect to the vibrational normal modes were obtained from the nuclear derivatives of the transition dipole moment, which is available after the vibrational analysis of the first excited state. The detailed procedure for the calculation of the transition dipole derivatives can be found in previous literature[17]. Sufficient vibrational quanta were included in the calculations to guarantee the convergence of the simulated fluorescence spectra. All of the obtained spectra were broadened by Lorentzian with full-width-at-half-maximum of 20 cm^{-1}.

S4.3 Electronic densities of the HOMO and LUMO as well as molecular geometrical configurations for the ground and excited states of a pentacene molecule

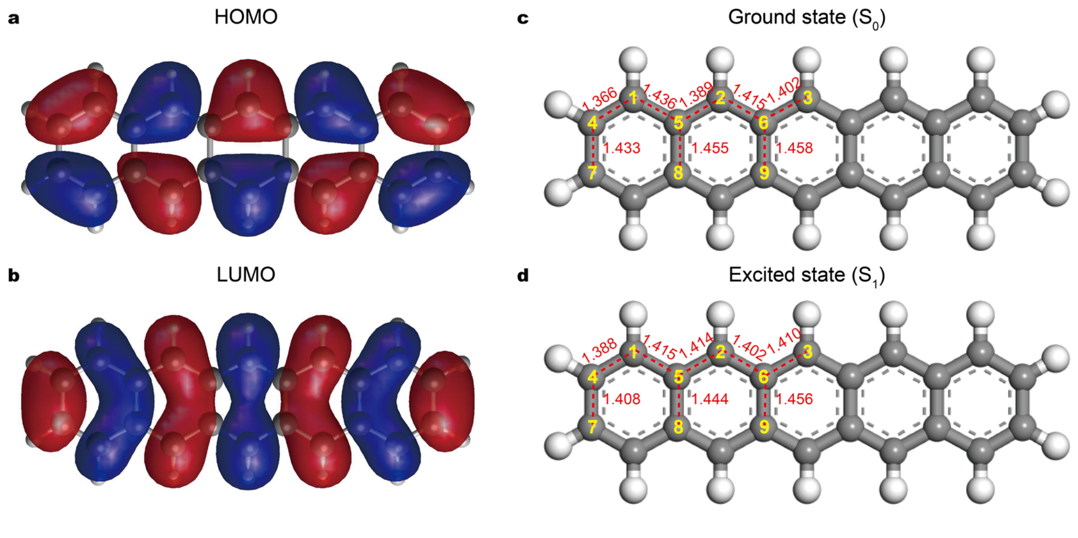

Supplementary Figure 3 | The electronic densities of molecular orbitals and the molecular

geometrical configurations for the ground and excited states. **a**, **b** Electronic densities of the HOMO and LUMO states, respectively, superimposed with a pentancene molecular skeleton. **c**, **d** The molecular geometrical configurations in ground and excited states, respectively. The calculated bond lengths between different carbon atoms are noted.

As shown in Supplementary Fig. 3, during an electronic transition from LUMO to HOMO, the bonding and anti-bonding characters between atoms will vary, and as a result, the nuclei are subjected to a change in Coulombic forces due to the redistribution of electronic charge. For example, as illustrated in Supplementary Fig. 3c and 3d, the bond length between carbon atoms 3 and 6 becomes shorter because an electron is transferred from an anti-bonding orbital into a bonding orbital; whereas the bond length between carbon atoms 2 and 6 becomes longer because an electron is transferred from a bonding orbital into an anti-bonding orbital[6].

S4.4 The calculation on the charge oscillations induced by molecular vibrations

In order to figure out how the molecular vibration change the electronic states, we have calculated the transition density difference ($\Delta\rho_{eg}$) corresponding to different vibration modes. The molecular structure is first optimized and used for the vibration calculation with Gaussian 16. The geometry of the pentacene molecule is then deformed along the atomic displacement $\Delta x_{k,n}$ for the *n*-th atom, which is determined by the related *k*-th vibration mode as $\Delta x_{k,n} = \pm\left(\phi_{k,n}^{x} / \sqrt{\mu_k}\right)Q_k^{v}$, where $\phi_{k,n}^{x}$ is the normalized displacement, μ_k is the reduced mass of the *k*-th mode. The amplitude in normal coordinate can be expressed as $Q_k^{v} = \sqrt{\left(v+1\right)\hbar / \left(2\omega_k\right)}$, where $\hbar$ is the Plank constant, ω_k is the vibration frequency of *k*-th mode, and *v* is the quantum number of vibrational level and set as *v* = 1 here. For the two most positively (Q_k^{+}) and negatively (Q_k^{-}) deformed configurations (two extreme molecular configurations), we can further calculate the wavefunctions for the associated molecular excited and ground states by the TDDFT method, and thus simulate the transition density via

$\rho_{eg}(\mathbf{r}) = \psi_e^*(\mathbf{r})\psi_g(\mathbf{r})$. Therefore, as illustrated in Supplementary Fig. 4, the charge oscillation induced by certain molecular vibration mode could be estimated from $\Delta\rho_{eg}(\mathbf{r}) \equiv (\partial\rho_{eg}(\mathbf{r})/\partial Q_k)\Delta Q_k \approx \left[\rho_{eg}^{Q_k^+}(\mathbf{r}) - \rho_{eg}^{Q_k^-}(\mathbf{r})\right]/2$, in which $\rho_{eg}^{Q_k^+}(\mathbf{r})$ and $\rho_{eg}^{Q_k^-}(\mathbf{r})$ stand for the transition density for the most positively (Q_k^+) and negatively (Q_k^-) deformed configurations, respectively. We would like to note that a similar treatment was used to simulate the conductance maps in scanning tunneling spectroscopy by taking into account the vibrationally mediated perturbations of molecular wave functions that go beyond the Franck-Condon model[18].

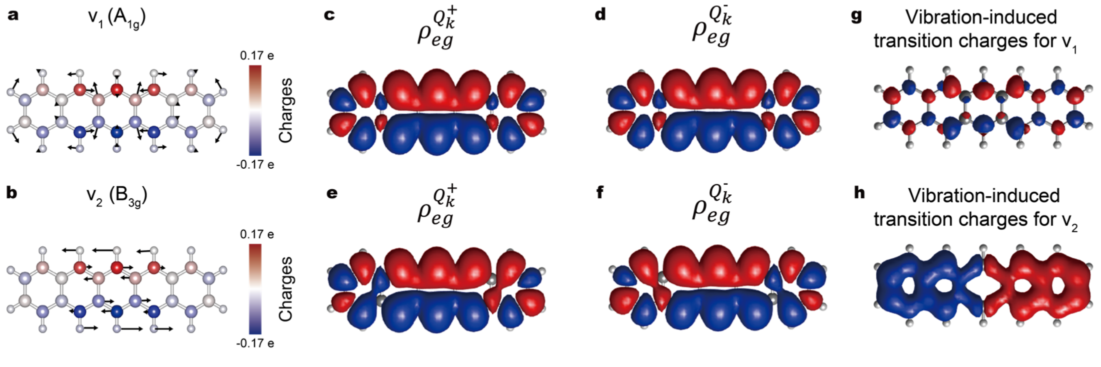

Supplementary Figure 4 | Calculated vibration-induced charge oscillation for v_1 and v_2 modes. **a**, **b** Schematics for the v_1 and v_2 vibrational modes, superimposed with the $S_1 \rightarrow S_0$ transition densities distributed for each atom by Hirschfield population analysis. **c**, **d**, Transition density for the most positively (Q_k^+) and negatively (Q_k^-) deformed configurations of v_1 mode. **e**, **f**, Transition density for the two most positively (Q_k^+) and negatively (Q_k^-) deformed configurations of v_2 mode. **g**, **h**, Calculated vibration-induced transition charges for v_1 and v_2 mode.

S4.5 Simulated photon images considering the HT or FC contribution alone

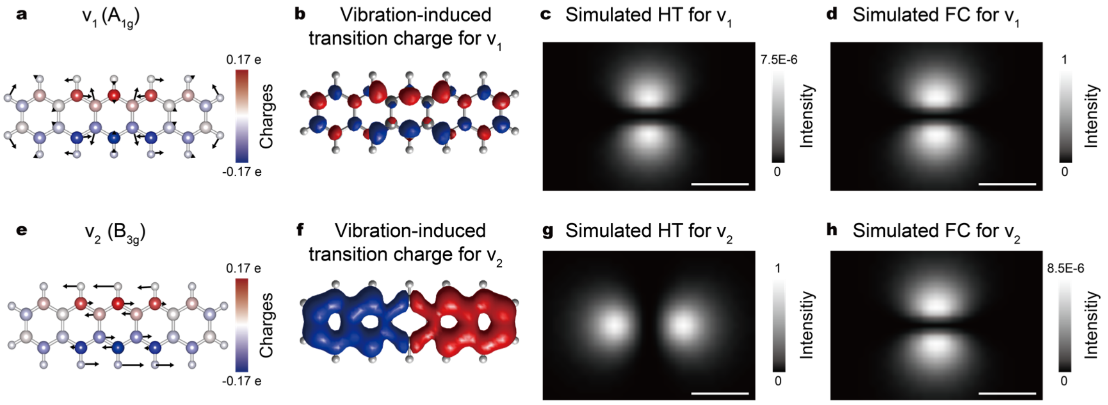

Supplementary Figure 5 | Simulated photon images considering the HT or FC contribution alone. **a** Schematics for the v_1 vibrational modes, superimposed with the $S_1 \rightarrow S_0$ transition distributed for each atom by Hirschfield population analysis. **b** Calculated vibration-induced transition charges for the v_1 mode. **c** and **d** Simulated photon images considering the contribution from the HT or FC term alone for the v_1 mode. The brightest point in **d** is normalized to 1. The brightest point in **c** is normalized based on the spectral peak ratio between the pure FC contribution and HT contribution calculated for the v_1 mode in main text Fig. 2a. **e** Schematics for the v_2 vibrational modes, superimposed with the transition density distributed for each atom by Hirschfield population analysis. **f** Calculated vibration-induced transition charges for the v_2 mode. **g** and **h** Simulated photon images considering the contribution from the HT or FC term alone for the v_2 mode.. The brightest point in **g** is normalized to 1. The brightest point in **h** is normalized based on the spectral peak ratio between the pure FC contribution and HT contribution calculated for the v_2 mode in main text Fig. 2a. Scale bars, 1 nm.

As illustrated in the relative magnitudes of the scale bars for Supplementary Fig. 5c and 5d as well as Supplementary Fig. 5g and 5h, for the v_1 mode, the HT contribution is negligible compared with the FC contribution. On the contrary, for the v_2 mode, the HT contribution overwhelms the FC contribution. Therefore, the features of the transition dipole and the associated two-spot pattern for v_1 mode are dominated by the FC contribution and are along the short axis, while for the v_2 mode, the features of the transition dipole and the associated two-spot pattern are dominated by the HT contribution and are along the long axis.

S4.6 Discussion on the selection rule based on the group theory

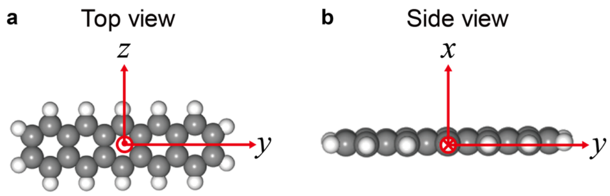

Supplementary Figure 6 | The molecular structure of pentacene with different views. a Top view. b Side view.

In this section, we would like to discuss on the selection rule of an electronic transition based on the group theory[19,20]. As illustrated in Supplementary Fig. 6, the point group of the pentacene molecule is D_{2h}. According to the calculation, the symmetries of the transition dipoles along the x, y and z axis are B_{3u}, B_{2u} and B_{1u}, respectively. Thus, for an electronic transition involving the irreducible representations of the electronic ground state (Γ_g), excited state (Γ_e), and the dipole ($\Gamma_{d,}$), the product of the transition $\Gamma_e \otimes \Gamma_d \otimes \Gamma_g$ from the ground state (A_g) and first excited state (B_{1u}) can be calculated as follows for different directions:

$$\begin{aligned} x &: B_{1u} \otimes B_{3u} \otimes A_g = B_{2g} \\ y &: B_{1u} \otimes B_{2u} \otimes A_g = B_{3g} \,, \\ z &: B_{1u} \otimes B_{1u} \otimes A_g = A_g \end{aligned} \tag{4}$$

Since an electronic transition is dipole-allowed only when $\Gamma_e \otimes \Gamma_d \otimes \Gamma_g$ contains the totally symmetric representation, the 0–0 transition is allowed and is only along the short axis (z direction). As shown in Eq. (3), because the FC term stems from the same electronic states of the 0–0 transition ($\boldsymbol{\mu}_{eg}(Q_0)$), the transition contribution from the FC term is also along the short axis. Thus, the transition dipole for the FC-dominated v_1 peak is oriented along the short axis.

As shown in Eq. (3) for the HT term, the symmetry analysis should take into account the additional symmetry of the vibration Γ_v. Accordingly, the HT term can contribute to the transition only when the product $\Gamma_e \otimes \Gamma_d \otimes \Gamma_g \otimes \Gamma_v$ contains the totally symmetric representation. Thus, the HT-term contribution can be allowed in x (y or z) direction for a molecular vibration with B_{2g} (B_{3g} or A_g) symmetry. Therefore, for the v_2 vibration of B_{3g} symmetry, the allowed HT contribution is along the long axis (y direction). In addition, according to our calculations, as shown in Supplementary Fig. 7, the vibration-induced transition is dominantly contributed from the vibration with B_{3g}

symmetry, in particular the v_2 mode, leading to a large vibration induced transition charges oscillating along the long axis (*y* direction). By contrast, the contributions from the other two directions for the vibrations with B_{2g} or A_g symmetry are about three orders of magnitude smaller than that from the *y* direction.

In this sense, the transition along the long axis direction is dipole-forbidden when only considering the symmetries of the electronic states, but can become allowed when taking into account the additional symmetry of the B_{3g} vibrations due to their perturbation to the electronic states, giving rise to vibration-induced emission.

We would like to note that, although the HT contributions along the vertical axis (*i.e.*, *x* direction) for the vertical B_{2g} molecular vibrations are about three orders of magnitude smaller than that along the long axis (*i.e.*, *y* direction) for the B_{3g} vibrations, we can still observe two weak but discernable emission peaks associated with these vertical vibrations. As shown in Supplementary Fig. 8, these two peaks are labeled as the v_5 mode at ~147 cm^{-1} and v_6 mode at ~874 cm^{-1} in the black curve acquired with the tip positioned at the pentacene center, which agree very well with the two strongest emission peaks induced by the B_{2g} vibrations in the calculated spectra. The observation of these two modes (v_5 and v_6 modes) in the STML measurements is probably due to the much stronger coupling of the vertical out-of-plane modes with the NCP field compared to the horizontal in-plane modes. In other words, the otherwise weak emission induced by the vertical B_{2g} vibrations is somehow "rescued" by the strong plasmonic field along the vertical axis. On the other hand, when the tip is positioned above the short and long axis directions of the pentacene molecule, no clear emission peaks induced by the B_{2g} vibrations are observed. The underlying physics is not completely clear yet, but might be associated with the tip-position dependent plasmon–molecule interaction at the vertical direction, which requires further experimental and theoretical studies.

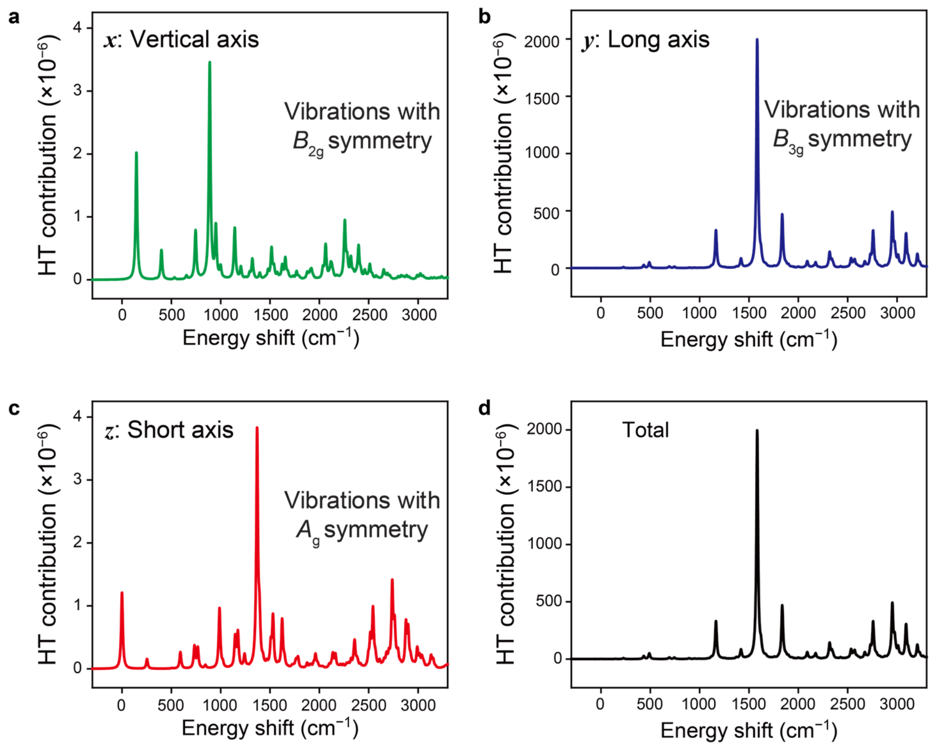

Supplementary Figure 7 | Calculated HT contributions in different directions for molecular vibrations with different symmetries. The total spectrum is the sum of the contributions from all three directions, which is almost the same as the spectrum in the *y* direction (long axis). The spectra were broadened by Lorentzian functions with full-width-at-half-maximum of 20 cm^{-1}.

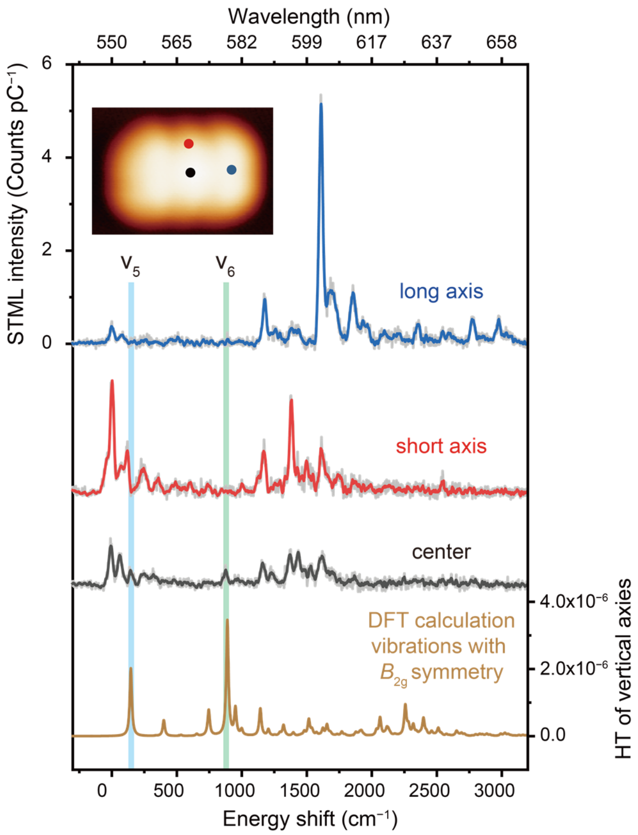

Supplementary Figure 8 | Analysis of the emission modes induced by the vibrations with B_{2g} symmetry. Three typical STML spectra (–3 V, 2 pA, 60 s) acquired at the positions marked in the inset. The raw data are plotted in grey lines. The calculated spectrum for the contributions from the HT term for the B_{2g} vibrations along the vertical axis (brown curve) are also plotted for comparison. The inset shows the STM image of a single pentacene molecule on 4ML-NaCl/Ag(100) (3.7 nm × 2.6 nm; –3 V, 2 pA).

S4.7 Discussion on the v_2 vibration-induced emission in the viewpoint of vibronic intensity borrowing

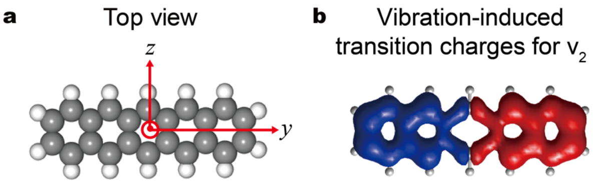

c

$S_i \rightarrow S_0$	μ_x	μ_y	μ_z	$E_i - E_1$(eV)
$S_2 \rightarrow S_0$	0.0000	0.0000	0.0000	1.0750
$S_3 \rightarrow S_0$	0.0000	0.0000	0.0000	1.4146
$S_4 \rightarrow S_0$	0.0000	**0.3630**	0.0000	1.5053

d

$S_1 \rightarrow S_i$	μ_x	μ_y	μ_z	$E_i - E_0$ (eV)
$S_1 \rightarrow S_2$	0.0000	**3.6637**	0.0000	2.7394
$S_1 \rightarrow S_3$	0.0000	**-0.9150**	0.0000	3.0790
$S_1 \rightarrow S_4$	0.0000	0.0000	0.0000	3.1697

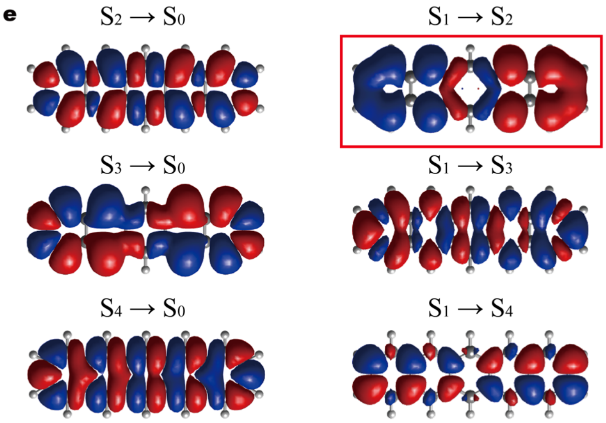

Supplementary Figure 9 | Simulated transition dipole moments and transition densities. a Molecular structure of a pentacene with a top view. **b** Calculated vibration-induced transition charges for the v_2 mode. **c** and **d** Calculated values of the transition dipoles between different electronic states along *x*, *y* and *z* directions and the corresponding energy differences. **e** Simulated different transition densities.

It should be noted that such vibration induced emission is often discussed in the literature using an intensity borrowing mechanism via the state mixing with other high-lying eigenstates[20-22], a quantum and more rigorous formulation, as expressed as follows:

$$
\begin{aligned}
\text{HT-term} &= \sum_k \left(\frac{\partial \langle \psi_g | \hat{\mu} | \psi_e \rangle}{\partial Q_k} \right)_0 \langle v_g | Q_k | v_e \rangle \\
&= \frac{1}{\hbar} \sum_k \left\{ \begin{array}{l} \sum_{i \neq e} \langle \psi_g^0 | \hat{\mu} | \psi_i^0 \rangle \dfrac{\langle \psi_i^0 | \left(\dfrac{\partial \hat{H}'}{\partial Q_k} \right)_0 | \psi_e^0 \rangle}{\omega_e - \omega_i} \\ + \sum_{i \neq g} \dfrac{\langle \psi_g^0 | \left(\dfrac{\partial \hat{H}'}{\partial Q_k} \right)_0 | \psi_i^0 \rangle}{\omega_g - \omega_i} \langle \psi_i^0 | \hat{\mu} | \psi_e^0 \rangle \end{array} \right\} \langle v_g | Q_k | v_e \rangle \\
&= \frac{1}{\hbar} \sum_k \left\{ \begin{array}{l} \sum_{i \neq e} \boldsymbol{\mu}_{ig}^0 (Q_0) \dfrac{h_{ie}^k}{\omega_e - \omega_i} \\ + \sum_{i \neq g} \dfrac{h_{gi}^k}{\omega_g - \omega_i} \boldsymbol{\mu}_{ei}^0 (Q_0) \end{array} \right\} \langle v_g | Q_k | v_e \rangle
\end{aligned} \tag{5}
$$

where g, e, and i represent the electronic ground state, first excited state, and the high-lying states, $\psi_g^0\,(\psi_e^0, \psi_i^0)$ represents the zero-order wavefunction of the corresponding electronic state at the equilibrium geometry (Q_0), $\hat{H}'$ represents the perturbation operator as a correction to the zero-order approximation, $\boldsymbol{\mu}_{ig}^0(Q_0)$ ($\boldsymbol{\mu}_{ei}^0(Q_0)$) represents the electronic transition dipole of $|i\rangle \to |g\rangle$ ($|e\rangle \to |i\rangle$) at the equilibrium geometry (Q_0), $\omega_e - \omega_i$ stands for the energy difference between states $|e\rangle$ and $|i\rangle$, while $\omega_g - \omega_i$ stands for the energy difference between states $|g\rangle$ and $|i\rangle$. As shown in Eq. (5), the HT term can gain the contribution from the dipoles of $\boldsymbol{\mu}_{ig}^0(Q_0)$ and $\boldsymbol{\mu}_{ei}^0(Q_0)$ through vibronic coupling, which seems to borrow intensities from neighboring electronic transitions.

As shown in Supplementary Fig. 9, the neighboring electronic transitions ($S_4 \to S_0$, $S_1 \to S_2$, and $S_1 \to S_3$) all have non-zero transition dipole moment along the long axis. Therefore, based on the dipole moments and energy differences, the v_2-vibration induced emission most probably borrows the contribution from the $S_1 \to S_2$ transition. Such an information can also be obtained based on the pattern similarities between the transition densities of $S_1 \to S_2$ transition and the v_2 vibration-induced transition charges. In other words, the v_2 vibration with B_{3g} symmetry is likely to distort the zero-order

electronic wavefunction of the S_0 state in a way to best resemble that of the S_2 state (*i.e.*, induce efficient mixing of the electronic ground state S_0 with the electronic excited state S_2), so that the v_2-vibration induced emission appears to gain large contributions from the $S_1 \rightarrow S_2$ transition. We would like to note that the model described in Supplementary Note 4, section 4.4 for the calculation of vibration-induced transition charges has implicitly accounted for the influences of all the contributing electronic states (including S_2).

S4.8 Discussions on the spatial influence of the combination tone mode on the electronic transition

In order to facilitate the discussion on the spatial influence of the overtone modes on the electronic transition, we can expand the vibrational wavefunctions in Eq. (3) into the commonly used multi-dimensional form. The multi-mode vibrational wavefunction can be expressed as $\left|v_g\right\rangle = \left|n_g^1, n_g^2, n_g^3, \cdots, n_g^N\right\rangle$, here N represents the total number of modes, n_g^k represents the quantum number of the k-th vibration of the ground state. Thus, the expression for the transition dipole in Eq. (3) can be re-written as

$$\begin{aligned}\boldsymbol{\mu}_{eg} = &\boldsymbol{\mu}_{eg}(Q_0)\left\langle n_g^1, n_g^2, n_g^3, \cdots, n_g^N \middle| n_e^1, n_e^2, n_e^3, \cdots, n_e^N\right\rangle + \\ &\sum\nolimits_k \left(\frac{\partial \boldsymbol{\mu}_{eg}}{\partial Q_k}\right)_0 \left\langle n_g^1, n_g^2, n_g^3, \cdots, n_g^N \middle| Q_k \middle| n_e^1, n_e^2, n_e^3, \cdots, n_e^N\right\rangle\end{aligned} \tag{6}$$

Our calculations show that the Duschinsky mode mixing effect plays only a minor role for the photon emission process due to the rigidity of the pentacene molecule, and dipole derivatives for the total symmetric modes (A_g symmetry modes) are very small. As a result, the transition dipoles for fundamental 0–1 transitions of both the v_2 and v_3 modes can then be expressed respectively as

$$\begin{aligned}\boldsymbol{\mu}_{eg,0-1_{v_2}} \approx &\boldsymbol{\mu}_{eg}(Q_0)\left\langle 0_g^1, 0_g^2, 0_g^3, \cdots, 1_g^{v_2}, \cdots, 0_g^N \middle| 0_e^1, 0_e^2, 0_e^3, \cdots, 0_e^{v_2}, \cdots, 0_e^N\right\rangle + \\ &\left(\frac{\partial \boldsymbol{\mu}_{eg}}{\partial Q_{v_2}}\right)_0 \left\langle 0_g^1, 0_g^2, 0_g^3, \cdots, 1_g^{v_2}, \cdots, 0_g^N \middle| Q_{v_2} \middle| 0_e^1, 0_e^2, 0_e^3, \cdots, 0_e^{v_2}, \cdots, 0_e^N\right\rangle\end{aligned} \tag{7}$$

and

$$\boldsymbol{\mu}_{eg,0-1_{v_3}} \approx \boldsymbol{\mu}_{eg}(Q_0)\left\langle 0_g^1, 0_g^2, 0_g^3, \cdots, 1_g^{v_3}, \cdots, 0_g^N \middle| 0_e^1, 0_e^2, 0_e^3, \cdots, 0_e^{v_3}, \cdots, 0_e^N \right\rangle + \left(\frac{\partial \boldsymbol{\mu}_{eg}}{\partial Q_{v_3}}\right)_0 \left\langle 0_g^1, 0_g^2, 0_g^3, \cdots, 1_g^{v_3}, \cdots, 0_g^N \middle| Q_{v_3} \middle| 0_e^1, 0_e^2, 0_e^3, \cdots, 0_e^{v_3}, \cdots, 0_e^N \right\rangle \quad (8)$$

For the HT-dominated v_2 mode, the integration of $\left\langle 0_g^1, 0_g^2, 0_g^3, \cdots, 1_g^{v_2}, \cdots, 0_g^N \middle| 0_e^1, 0_e^2, 0_e^3, \cdots, 0_e^{v_2}, \cdots, 0_e^N \right\rangle$ is negligible due to the orthogonality of the wavefunctions $0_e^{v_2}$ and $1_g^{v_3}$, and thus, Eq. (7) can be approximated as

$$\boldsymbol{\mu}_{eg,0-1_{v_2}} \approx \left(\frac{\partial \boldsymbol{\mu}_{eg}}{\partial Q_{v_2}}\right)_0 \left\langle 0_g^1, 0_g^2, 0_g^3, \cdots, 1_g^{v_2}, \cdots, 0_g^N \middle| Q_{v_2} \middle| 0_e^1, 0_e^2, 0_e^3, \cdots, 0_e^{v_2}, \cdots, 0_e^N \right\rangle \quad (9)$$

where the term $\left(\frac{\partial \boldsymbol{\mu}_{eg}}{\partial Q_{v_2}}\right)_0$ is mainly responsible for the spatial influence of the v_2 mode on the electronic transition.

For the v_3 mode with A_g symmetry, similar to the analysis on v_1 mode in Supplementary Note 4, section 4.6, the corresponding vibronic peak is dominantly contributed from the FC term and the term $\left(\frac{\partial \boldsymbol{\mu}_{eg}}{\partial Q_{v_3}}\right)_0$ is negligible. Thus, Eq. (8) can be approximated as

$$\boldsymbol{\mu}_{eg,0-1_{v_3}} \approx \boldsymbol{\mu}_{eg}(Q_0)\left\langle 0_g^1, 0_g^2, 0_g^3, \cdots, 1_g^{v_3}, \cdots, 0_g^N \middle| 0_e^1, 0_e^2, 0_e^3, \cdots, 0_e^{v_3}, \cdots, 0_e^N \right\rangle \quad (10)$$

The transition dipole for the 0–2 transition of the v_4 combination mode can be expressed as

$$\begin{aligned}\boldsymbol{\mu}_{eg,0-2_{v_4}} \approx\ & \boldsymbol{\mu}_{eg}(Q_0)\left\langle 0_g^1, 0_g^2, 0_g^3, \cdots, 1_g^{v_3}, \cdots, 1_g^{v_2}, \cdots, 0_g^N \middle| 0_e^1, 0_e^2, 0_e^3, \cdots, 0_e^{v_3}, \cdots, 0_e^{v_2} \cdots, 0_e^N \right\rangle \\ & + \left(\frac{\partial \boldsymbol{\mu}_{eg}}{\partial Q_{v_2}}\right)_0 \left\langle 0_g^1, 0_g^2, 0_g^3, \cdots, 1_g^{v_3}, \cdots, 1_g^{v_2}, \cdots, 0_g^N \middle| Q_{v_2} \middle| 0_e^1, 0_e^2, 0_e^3, \cdots, 0_e^{v_3}, \cdots, 0_e^{v_2} \cdots, 0_e^N \right\rangle \\ & + \left(\frac{\partial \boldsymbol{\mu}_{eg}}{\partial Q_{v_3}}\right)_0 \left\langle 0_g^1, 0_g^2, 0_g^3, \cdots, 1_g^{v_3}, \cdots, 1_g^{v_2}, \cdots, 0_g^N \middle| Q_{v_3} \middle| 0_e^1, 0_e^2, 0_e^3, \cdots, 0_e^{v_3}, \cdots, 0_e^{v_2} \cdots, 0_e^N \right\rangle\end{aligned} \quad (11)$$

Considering the negligible $\left(\frac{\partial \boldsymbol{\mu}_{eg}}{\partial Q_{v_3}}\right)_0$ term and the orthogonality of the wavefunctions $0_e^{v_2}$ and $1_g^{v_2}$, Eq. (11) can be approximated as

$$\boldsymbol{\mu}_{eg,0-2_{v_4}} \approx \left(\frac{\partial \boldsymbol{\mu}_{eg}}{\partial Q_{v_2}}\right)_0 \left\langle 0_g^1, 0_g^2, 0_g^3, \cdots, 1_g^{v_3}, \cdots, 1_g^{v_2}, \cdots, 0_g^N \middle| Q_{v_2} \middle| 0_e^1, 0_e^2, 0_e^3, \cdots, 0_e^{v_3}, \cdots, 0_e^{v_2} \cdots, 0_e^N \right\rangle \tag{12}$$

As shown in Eq. (12), the term $\left(\frac{\partial \boldsymbol{\mu}_{eg}}{\partial Q_{v_2}}\right)_0$ is mainly responsible for the spatial influence of the v_4 mode on the electronic transition, which would lead to a vibration induced transition charge oscillations similar to the v_2 mode. Thus, the combined influence of two simultaneously excited fundamental vibrations (v_2 and v_3 modes) on the electronic transition is very similar to that of the v_2 mode. Such expectation is consistent with the experimental observation that the two-spot pattern of the v_4 (v_4') mode is along the long axis of pentacene (perdeuterated pentacene), as shown in the inset of main-text Fig. 4c.

Supplementary Note 5 STML spectra from a single perdeuterated pentacene molecule and the energy-resolved spectroscopic images

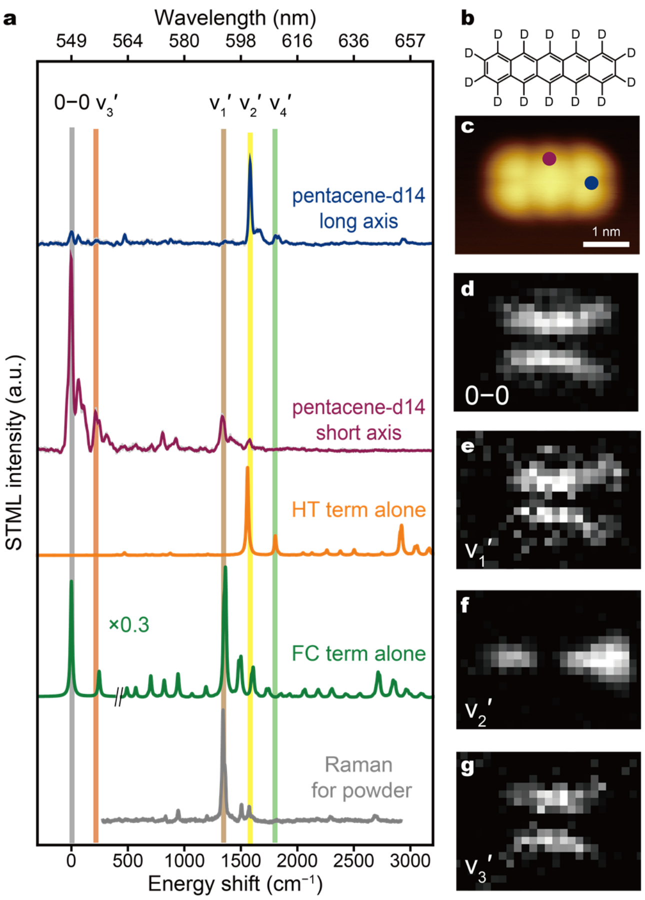

Supplementary Figure 10 | STML spectra from a perdeuterated pentacene molecule and energy-resolved spectroscopic images. a STML spectra (–3 V, 2 pA, 600 s) from a single perdeuterated pentacene molecule acquired at both the long axis and short axis as marked in the STM image of **c**. The raw data are plotted in grey lines. The DFT calculated spectra for the pure FC contribution (green line) and HT contribution (orange line) are plotted for comparison. In addition, the Raman spectrum acquired from a perdeuterated pentacene powder sample at room temperature (excitation laser wavelength: 633 nm) is also plotted (gray curve). **b** and **c** The molecular structure and the STM image of a perdeuterated pentacene molecule (4 nm × 3 nm; –3 V, 2 pA). **d**–**g**, Energy-resolved spectroscopic images for the 0–0 peak (0±7 cm^{-1}, **d**), the v_1' mode (1335±6 cm^{-1}, **e**), the v_2' mode (1582±6 cm^{-1}, **f**), and the v_3' mode (221±6 cm^{-1}, **g**). Conditions: 4 nm × 3 nm; –3 V, 2 pA; 5 s per pixel.

Supplementary Fig. 10 shows the excitation position-dependent STML spectra from

a single perdeuterated pentacene molecule. The electroluminescence peak with the highest emission energy is at ~548.7 nm, which is assigned to the 0–0 emission peak of a single perdeuterated pentacene and is very close to that of a single pentacene (~549.7 nm). The vibronic peaks with lower energies in the STML spectra can then be expressed as the frequency shifts with respect to the 0–0 peak, which agree well with the vibronic features in the DFT calculated spectra (orange and green curve) and the Raman spectrum from a powder sample (gray curve). As shown in Supplementary Fig. 9d–g, the photon images of the 0–0 peak, the v_1' mode (~1335 cm^{-1}) and v_3' mode (~221 cm^{-1}) show the two-spot patterns along the short axis, while the photon image of the v_2' mode (~1582 cm^{-1}) shows the two-spot pattern along the long axis, which are all similar to the patterns of their counterpart modes of a single pentacene.

Supplementary References

1. Chen, G., *et al.* Spin-triplet-mediated up-conversion and crossover behavior in single-molecule electroluminescence. *Phys. Rev. Lett.* **122**, 177401 (2019).
2. Kimura, K., *et al.* Selective triplet exciton formation in a single molecule. *Nature* **570**, 210-213 (2019).
3. Born, M., Oppenheimer, R. Zur quantentheorie der molekeln. *Ann. Phys. (Leipzig)* **389**, 457-484 (1927).
4. Franck, J., Dymond, E. Elementary processes of photochemical reactions. *Trans. Faraday Society* **21**, 536-542 (1926).
5. Condon, E. U. Nuclear motions associated with electron transitions in diatomic molecules. *Phys. Rev.* **32**, 858-872 (1928).
6. Atkins, P., Friedman, R. *Molecular quantum mechanics* (Oxford University Press, Oxford, 2011).
7. Barone, V., Bloino, J., Biczysko, M. Vibrationally-resolved electronic spectra in GAUSSIAN 09. *GAUSSIAN 09 Revision A* **2**, 1-20 (2009).
8. Azumi, T., Matsuzaki, K. What does the term "vibronic coupling" mean? *Photochem. Photobiol.* **25**, 315-326 (1977).
9. Herzberg, G., Teller, E. Schwingungsstruktur der elektronenübergänge bei mehratomigen molekülen. *Z. Phys. Chem.* **21**, 410-446 (1933).
10. Lee, C., Yang, W., Parr, R. G. Development of the Colle-Salvetti correlation-energy formula into a functional of the electron density. *Phys. Rev. B* **37**, 785-789 (1988).
11. Stephens, P. J., Devlin, F. J. Ab initio calculation of vibrational absorption and circular dichroism spectra using density functional force fields. *J. Phys. Chem.* **98**, (1994).
12. Becke, A. D. Density-functional thermochemistry. III. The role of exact exchange. *J. Chem. Phys.* **98**, (1993).
13. Frisch, M. J., *et al.*, Gaussian 16 Rev. C.01, 2016.

14. Aidas, K., *et al.* The Dalton quantum chemistry program system. *Wiley Interdiscip. Rev.: Comput. Mol. Sci.* **4**, 269-284 (2014).
15. Dalton, a molecular electronic structure program, Release Dalton2017.alpha, see http://daltonprogram.org.
16. Tian, G. J., Duan, S., Hua, W., Luo, Y. DynaVib Version 1.0. *Royal Institute of Technology, Sweden*, (2012).
17. Qiu, F. F., Song, C., Li, L., Wei, Y., Tian, G. J. First-principles study on vibrationally resolved fluorescence of fused 5,15-(diphenyl)-10,20-(dibromo)porphyrin molecule. *J. Chem. Phys.* **149**, 074312 (2018).
18. Reecht, G., Krane, N., Lotze, C., Zhang, L., Briseno, A. L., Franke, K. J. Vibrational excitation mechanism in tunneling spectroscopy beyond the Franck-Condon model. *Phys. Rev. Lett.* **124**, 116804 (2020).
19. McHale, J. L. *Molecular spectroscopy* (CRC Press, Boca Raton, 2017).
20. Kuhnke, K., Große, C., Merino, P., Kern, K. Atomic-scale imaging and spectroscopy of electroluminescence at molecular interfaces. *Chem. Rev.* **117**, 5174-5222 (2017).
21. Long, D. A. *The Raman effect: A unified treatment of the theory of Raman scattering by molecules* (John Wiley & Sons Ltd., Chichester, 2002).
22. Doppagne, B., *et al.* Vibronic spectroscopy with submolecular resolution from STM-induced electroluminescence. *Phys. Rev. Lett.* **118**, 127401 (2017).
