# Supplementary figures and images for: Probing intramolecular vibronic coupling through vibronic-state imaging
Source: Nat Commun. 2021 Feb 24;12:1280. doi: 10.1038/s41467-021-21571-z (PMC7904785; doi:10.1038/s41467-021-21571-z)

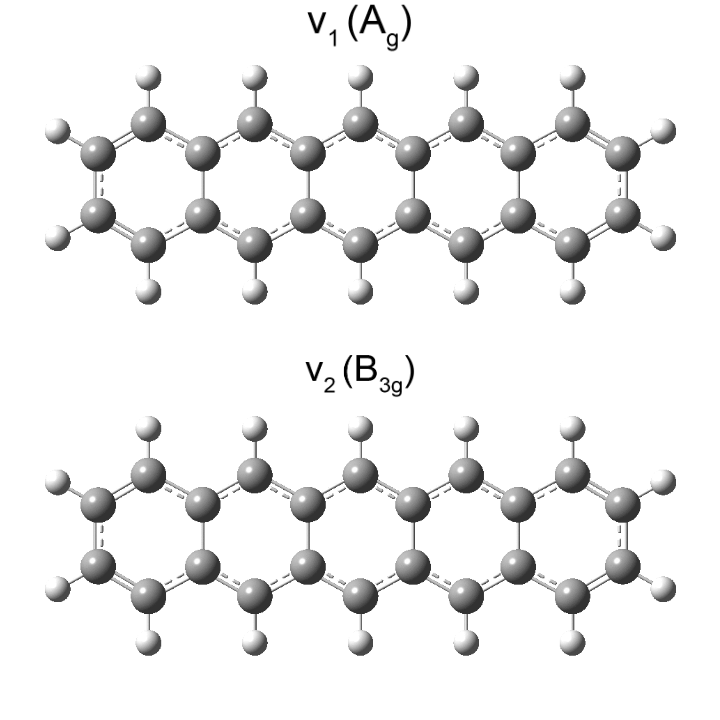

Supplement: Supplementary file 2 — Supplementary Movie 1 [file 41467_2021_21571_MOESM2_ESM.gif]
