# Supplementary material for: Probing intramolecular vibronic coupling through vibronic-state imaging
Source: Nat Commun. 2021 Feb 24;12:1280. doi: 10.1038/s41467-021-21571-z (PMC7904785; doi:10.1038/s41467-021-21571-z)
Supplement: Supplementary file 3 — Description of Additional Supplementary Files [file 41467_2021_21571_MOESM3_ESM.docx]

File Name: Supplementary Movie 1
Description:
Cartons for v_1_ and v_2_ vibrational modes.
